# Latent profile analysis of parental burnout among parents of children with and without autism spectrum disorder

**DOI:** 10.3389/fpsyg.2025.1581321

**Published:** 2025-04-07

**Authors:** Shuyu Liu, Dehua Wu, Jibo Li, Huazhan Yin

**Affiliations:** ^1^Guangdong Provincial Key Laboratory of Development and Education for Special Needs Children, Lingnan Normal University, Zhanjiang, China; ^2^School of Psychology, Hunan Normal University, Changsha, China

**Keywords:** latent profile analysis, parental burnout, parental caregivers, children, autism spectrum disorder

## Abstract

**Background:**

Parental burnout is an emerging global focus on parental mental health and parenting practices. However, parental burnout among parents of children with autism spectrum disorder (ASD) in China remains largely unexplored.

**Objective:**

This study aimed to identify differences in parental burnout severity and patterns between Chinese parents raising children with and without ASD, while exploring key sociodemographic factors contributing to these disparities.

**Methods:**

In total, 1,048 Chinese parents (including 487 parents of children with ASD) were recruited to take part in an online survey. The participants completed the Chinese version of Brief Parental Burnout Assessment and Brief Demographic Questionnaire. For data analysis, Latent Profile Analysis (LPA) was employed to identify distinct burnout profiles, followed by multivariate logistic regression to examine the association between sociodemographic factors and profile membership.

**Results:**

The LPA revealed distinct classification patterns between the two groups: parents of children with ASD demonstrated three subgroups (Low, Medium, and High parental burnout profiles), while parents of children without ASD exhibited only two subgroups (Low, Medium parental burnout profiles). Multivariate logistic regression analysis found that mothers, parents with two or more children, parents with younger children, and parents of children with severe ASD were associated with high parental burnout profiles.

**Conclusion:**

Parents of children with ASD were more likely to experience higher levels of parental burnout. It highlights an urgency for targeted interventions to different burnout subgroups of parents having children with ASD.

## Introduction

Acting as a chronic disorder linked to parenting stress, parental burnout is considered a manifestation of the parents’ poor mental health and negative parenting practice (Mikolajczak et al., 2021; Yu, 2021). It encompasses feelings of fatigue in parenting, emotional detachment from children, and a sense of inadequacy in fulfilling parental duties (Mikolajczak et al., 2019). Over the past few years, parental burnout has drawn significant interest among researchers internationally (Griffith, 2020; Mikolajczak et al., 2018a); however, few studies have focused on this theme among parents who raise children with disabilities in China (Wang and Wang, 2021).

Autism spectrum disorder (ASD) is a neurodevelopmental disorder characterized by challenges in social communication and interaction, as well as restricted interests and repetitive behaviors (American Psychiatric Association, 2013). Recognized as a global public health concern, the prevalence rate of ASD in China is 0.7 in 2022 (Zeidan et al., 2022). According to the relationship satisfaction model in the context of children with ASD (Sim et al., 2016), raising a child with ASD can be associated with unique challenges, which often contribute to high levels of stress and burnout among parents, making it an urgent issue that needs to be addressed. While social support is of significant importance to the well-being of those families (Liu et al., 2024). Given the high prevalence of ASD and the significant challenges faced by families, understanding the factors that contribute to parental burnout is critical for developing effective support systems and improving family well-being.

Latent profile analysis (LPA) is a person-centered statistical tool that relies on latent category variables to interpret the relationships between external continuous variables, ensuring local independence among explicit variables (Collins and Lanza, 2009). In academic practice, this technique has found widespread application in differentiating heterogeneous groups within the realms of social sciences, mental health research, and behavioral studies (Burns et al., 2022; Jiang et al., 2023). Suárez et al. (2022) carried out a LPA and discovered four potential parental burnout profiles in Spanish parents: Low Parental Burnout Profile (LPBP), Medium Parental Burnout Profile (MPBP), High Parental Burnout Profile (HPBP), and Very High Parental Burnout Profile (VHPBP). The study provided significant value for understanding the heterogeneity of parental burnout in Western contexts. However, prior research has extensively explored parental burnout in Western, individualist contexts, less attention has been paid to how cultural values in Eastern, collectivist societies may shape the experience of burnout (Roskam et al., 2021). With a strengthened social network of mutual aid and solidarity around families (Szczygieł et al., 2020), the prevalence of parental burnout in collectivistic countries (i.e., China) is lower. In addition, researchers have found out there are differences between burnout of parents who raise children with and without disabilities (Ardic, 2020; Kütük et al., 2021). Thus, one of the primary aims of this study is to use LPA to identify differences in parental burnout latent profiles between Chinese parents raising children with and without ASD.

The balance between risks and resources theory (Mikolajczak and Roskam, 2018) points out that sociodemographic factors that contain important family information have great effects on parental burnout (Mikolajczak et al., 2018b). As is known, the gender of parents, family income, parents’ education level, number of children, gender of child, severity of ASD, and age of the child are key sociodemographic factors (Le Vigouroux and Scola, 2018; Chen et al., 2022; Ardic and Olcay, 2021). The systematic inclusion of sociodemographic determinants as predictive parameters for subgroup offers valuable insights for supports addressing the unique needs of various demographic populations affected by parental burnout. Thus, the other aim of this study is to explore the predictive effects of key sociodemographic factors on parental burnout profiles.

Through systematic examination of prior studies, we have established two testable hypotheses for investigation:

*Hypothesis 1*: Parents of children with and without ASD exhibit different latent profile classifications in terms of parental burnout.

*Hypothesis 2*: Sociodemographic factors (gender of parents, family income, parents’ education level, number of children, gender of child, severity of ASD, and age of the child) have predictive effects on these latent classifications.

## Methods

### Participants

Using convenience sampling techniques, we distributed an online survey to parents of children aged 3–17, from schools in the Guangdong and Hunan Provinces of China. The teachers helped us send the network link to the parents in March and December 2022 via WeChat. On the first page of the questionnaire, the purpose of the survey was explained, and the relevant information was kept confidential. After obtaining informed consent, the participants completed a survey questionnaire. A total of 1,048 valid data were obtained, including 487 from parents raising at least a child with ASD and 561 from parents of typically developed children. Those children with ASD were students in special education schools, who obtained a diagnosis certificate from a specialist doctor before they could be enrolled in special education schools. These certificates typically include a severity classification (e.g., mild, moderate, severe) based on clinical evaluation. In this study, parents reported their child’s severity of ASD based on the classification. Those typically developed children were students in general primary or secondary schools. We excluded participants based on the following criteria: (a) parents who were not the primary caregivers at home, (b) cases where the severity of the ASD diagnosis was not clearly confirmed, and (c) parents who provided overly uniform or patterned responses to the questionnaire items. The parents who participated in the survey were aged 21–58 (*M_age_* = 36.49, *SD* = 6.55). Table 1 shows the demographic information.

**Table 1:** Demographic information of the parents and children.

Parents of children with ASD (*N* = 487)				Parents of children without ASD (*N* = 561)			
		*n*	*%*			*n*	*%*
Gender of parents	Father	179	36.76%	Gender of parents	Father	208	37.08%
	Mother	308	63.24%		Mother	353	62.92%
Education of parents	Compulsory school	74	15.20%	Education of parents	Compulsory school	214	38.15%
	High school	146	29.98%		High school	155	27.63%
	Some college	202	41.48%		Some college	100	17.82%
	Bachelor’s degree	65	13.35%		Bachelor’s degree	92	16.40%
Family monthly income	0–2,999 RMB	85	17.45%	Family monthly income	0–2,999 RMB	110	19.61%
	3,000–5,999 RMB	131	26.90%		3,000–5,999 RMB	176	31.37%
	6,000–8,999 RMB	133	27.31%		6,000–8,999 RMB	124	22.10%
	Over 9,000 RMB	138	28.34%		Over 9,000 RMB	151	26.92%
Number of children	One	223	45.79%	Number of children	One	111	19.79%
	Two or more	264	54.21%		Two or more	450	80.21%
Gender of the child	Boy	356	73.10%	Gender of the child	Boy	297	52.94%
	Girl	131	26.90%		Girl	264	47.06%
Age of the child	Age 3–6	192	39.43%	Age of the child	Age 3–6	129	22.99%
	Age 7–12	237	48.67%		Age 7–12	278	49.56%
	Age 13–17	58	11.91%		Age 13–17	154	27.45%
Severity of ASD	Mild	220	45.17%				
	Moderate	182	37.37%				
	Severe	85	17.45%				

This study was conducted under the sustained ethical supervision of the Institutional Ethics Review Board at the researcher’s academic affiliation, having obtained full ethical clearance before commencement.

### Measures

#### Brief Demographic Questionnaire

Brief Demographic Questionnaire was administered including the gender of parents, monthly family income, education level of parents, gender of the child, number of children, and severity of ASD.

#### Brief Parental Burnout Assessment

The Chinese version of the Brief Parental Burnout Assessment (Wang et al., 2021) was used to measure parental burnout. This is a 7-item scale (e.g., “I feel completely run down by my role as a parent”). Responses were evaluated using a seven-point Likert scale. Here, a rating of 0 corresponded to “never,” and a rating of 6 stood for “every day.” An increase in the scores on this scale was an indication of more severe parental burnout. The Cronbach’s alpha coefficient of the scale in this study was 0.928.

### Data analysis

Mplus 8.3 statistical software was employed to evaluate the latent profile of parental burnout to determine the latent category and distribution of parental burnout. Using the satisfaction with the seven items of Brief Parental Burnout Assessment as indicator variables, LPA with 1 to 5 categories were fitted to estimate and compare the burnout of parents of children with and without ASD. The fitting information commonly used in LPA includes the Akaike information criterion (AIC), Bayesian information criterion (BIC), adjusted Lo–Mendell–Rubin corrected likelihood ratio (LMRT), bootstrap-based likelihood ratio test index, and average information quantity (Entropy) (Lo et al., 2001; Muthén and Muthén, 2000; Peel and McLachlan, 2000). A model with higher Entropy, lower AIC and BIC, and significant LMRT indicates a higher degree of fit (Miller et al., 2009; Muthén and Muthén, 2007; Xian et al., 2005).

Statistical software (SPSS 21.0) was used to conduct independent sample t-tests and multivariate logistic regression analysis. Multivariate logistic regression analysis was used to investigate the influence of sociodemographic variables on the latent profiles of parental burnout.

## Results

### Descriptive statistics and comparison of parental burnout of the two groups

The independent samples t-test revealed that burnout scores of parents having children with ASD were significantly higher than those of typically developing children (Table 2).

**Table 2:** T-test of parental burnout of the two groups of parents.

Participant Groups	*N*	*M*	*SD*	*t*
Parents of children with ASD	487	19.17	9.55	12.28^***^
Parents of children without ASD	561	12.51	7.75	

^***^p < 0.001.

### Latent profile analysis of parental burnout

A latent profile model was established based on the average value of each parental burnout item and five latent category models were extracted. Table 3 showed the results of the model fitting information.

**Table 3:** Latent profile model fitting indicators.

Participant Groups	Category	*AIC*	*BIC*	*aBIC*	*Entropy*	*LMR*-LRT	*BLRT*	Class probability (%)
Parents of children with ASD	1	12983.42	12997.62					1
	2	11217.30	11309.44	11239.61	0.93	<0.001	<0.001	55.24/44.76
	3	10615.73	10741.38	10646.18	0.95	0.002	<0.001	50.10/42.90/8.00
	4	10468.59	10627.74	10507.13	0.91	0.100	<0.001	47.84/32.24/16.22/3.70
	5	10382.51	10575.17	10429.17	0.88	0.625	<0.001	39.01/18.48/32.85/6.16/3.49
Parents of children without ASD	1	13754.80	13815.41	13770.97				1
	2	11813.94	11909.19	11839.36	0.98	<0.001	<0.001	84.67/15.33
	3	11368.07	11497.96	11402.73	0.97	0.072	<0.001	80.22/14.97/4.81
	4	11111.86	11276.36	11155.76	0.98	0.145	<0.001	79.50/11.77/3.92/4.81
	5	10900.58	11099.75	10953.72	0.97	0.434	<0.001	74.40/6.60/3.74/9.45/4.81

AIC, Akaike information criterion; BIC, Bayesian information criterion; LMRT, Lo–Mendell–Rubin corrected likelihood ratio; BLRT, bootstrap-based likelihood ratio test index.

**Figure 1:**
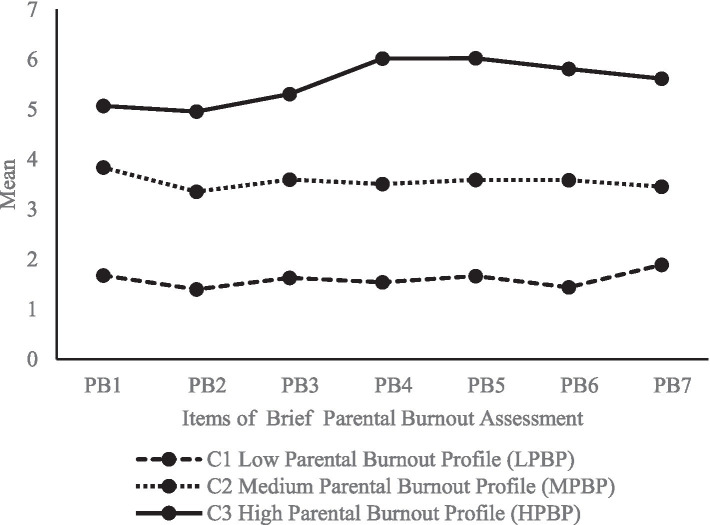
Estimated conditional means for latent profile of parental burnout among parents of children with ASD.

For parents of children with ASD, the three-class model was selected as it presents better fit statistics than the two-or four-class models. The AIC, BIC, and ABIC statistics of the three-class model were lower than those of the two-class model. Furthermore, the LMRT-test of the three-class model was statistically significant (*p* < 0.01), suggesting that this model fit the data better than the two-class model. Similarly, none of the three classes had fewer than 5% individuals, and the entropy index was 0.95. However, although the AIC, BIC, and ABIC statistics of the four- and five-class models were lower, the LMRT test results were not statistically significant (*p* > 0.01). In addition, the conditional probability of the three latent profiles on the seven items were obtained (Figure 1), where C1, C2, and C3 had different levels on the seven items of the Brief Parental Burnout Assessment. The conditional mean of C1 (50.10%) was the lowest. The conditional mean of C3 (8.00%) was higher than that of the other two classes (C1, C2) for all seven items; that is, this class suffered from a higher degree of parental burnout. C2 (41.90%) was between those of C1 and C3. Therefore, C1, C2, and C3 were named LPBP, MPBP, and HPBP, respectively. More than half of the parents raising children with ASD in the total sample were in MPBP or HPBP.

**Figure 2:**
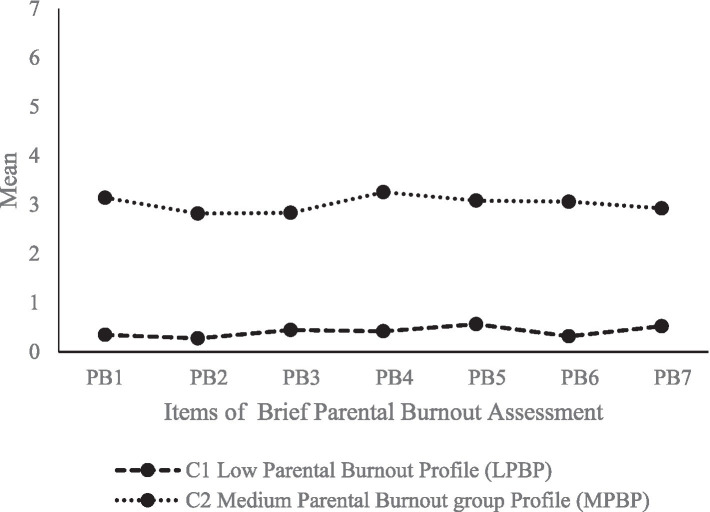
Estimated conditional means for latent profile of parental burnout among parents of children without ASD.

For parents of children without ASD, the two-class model was selected as it presented better fit statistics than the other models (Table 3). Furthermore, the conditional probabilities of the two latent profiles for the seven items were obtained, as shown in Figure 2. The conditional mean of C1 (84.67%) was lower and the conditional mean of C2 (15.33%) was higher than that of C1 for all seven items; that is, the class suffered from a medium degree of parental burnout. Therefore, C1 and C2 were named LPBP and MPBP, respectively.

### Analysis of sociodemographic characteristics for profiles of parental burnout

Multivariate logistic regression analysis was conducted to explore the predictive effect of sociodemographic factors on parental burnout latent profiles. Latent profiles are the dependent variable and sociodemographic factors (gender of parents, monthly family income, education level of parents, number of children, gender of the child, age of the child, and severity of ASD) are independent variables, with HPBP as the reference category among parents of children with ASD and MPBP as the reference category among parents of children without ASD. The OR reveals the effect of each covariate variable on the likelihood of a profile. The results of multivariate logistic regression analysis are shown in Tables 4, 5.

**Table 4:** Odd ratio reflecting effects of sociodemographic variables on latent profiles of parental burnout among parents of children with ASD.

Reference group		Low parental burnout profile (LPBP)			Medium parental burnout profile (MPBP)		
		*β*	*p*	*OR[95%CI]*	*β*	*p*	*OR[95%CI]*
Gender of parents							
Father	Mother	−0.89	0.02	0.41[0.19, 0.88]	0.21	0.58	1.23[0.59, 2.57]
Education of parents							
Bachelor’s degree	Secondary school	−0.42	0.59	0.66[0.14, 3.05]	−0.27	0.75	0.76[0.15, 3.91]
	High school	−0.46	0.50	0.63[0.17, 2.37]	0.75	0.28	2.13[0.54, 8.44]
	Some college	−0.78	0.21	0.46[0.13, 1.57]	0.77	0.25	2.15[0.59, 7.89]
Family monthly income							
Over 9,000 RMB	0–2,999 RMB	0.39	0.61	1.47[0.33, 6.50]	0.49	0.52	1.64[0.36, 7.37]
	3,000–5,999 RMB	−0.90	0.11	0.41[0.14, 1.21]	−0.27	0.62	0.76[0.26, 2.24]
	6,000–8,999 RMB	−0.48	0.38	0.62[0.21, 1.82]	−0.45	0.41	0.64[0.22, 1.87]
Number of children							
One	Two or more	−0.99	0.01	0.37[0.17, 0.82]	−0.35	0.38	0.71[0.33, 1.52]
Gender of the child							
Boy	Girl	0.14	0.75	1.15[0.49, 2.73]	−0.21	0.62	0.81[0.35, 1.88]
Age of the child							
Age 13–18	Age 3–6	−0.65	0.32	0.52[0.15, 1.88]	0.11	0.87	1.12[0.31, 4.09]
	Age 7–12	−0.02	0.97	0.98[0.29, 3.31]	−0.07	0.91	0.93[0.27, 3.26]
Severity of ASD							
Severe	Mild	2.84	<0.01	17.19[5.92, 49.94]	1.59	<0.01	4.93[1.74, 13.94]
	Moderate	1.12	0.01	3.07[1.27, 7.41]	1.29	<0.01	3.62[1.55, 8.48]

**Table 5:** Odd ratio reflecting effects of sociodemographic variables on latent profiles of parental burnout among parents of children without ASD.

Reference group		Low Parental Burnout Profile (LPBP)		
		*β*	*p*	*OR[95%CI]*
Gender of parents				
Father	Mother	0.85	0.76	1.09[0.63, 1.88]
Education of parents				
Bachelor’s degree	Secondary school	−0.03	0.95	0.97[0.40, 2.36]
	High school	−0.15	0.73	0.86[0.36, 2.04]
	Some college	0.13	0.78	1.14[0.45, 2.91]
Family monthly income				
Over 9,000 RMB	0–2,999 RMB	−2.08	<0.01	0.13[0.05, 0.32]
	3,0005,999 RMB	−1.65	<0.01	0.19[0.08, 0.47]
	6,000–8,999 RMB	−1.10	0.02	0.33[0.13, 0.83]
Number of children				
One	Two or more	0.12	0.72	1.12[0.60, 2.09]
Gender of the child				
Boy	Girl	0.41	0.87	1.04[0.64, 1.70]
Age of the child				
Age 13–18	Age 3–6	−0.90	0.01	0.41[0.20, 0.83]
	Age 7–12	0.33	0.30	1.38[0.74, 2.57]

For parents raising ASD children, mothers, families with two or more children, parents of children aged 3–6 years, and parents of children with severe ASD were more likely to be in the HPBP (Table 4). For parents raising normal children, a monthly family income of 9,000 RMB or above and parents of children over 6 years of age were more likely to be in the LPBP (Table 5).

## Discussion

### Latent profile of parental burnout

As far as we know, this study found for the first time in a Chinese sample that parents of children with and without ASD differ in their parental burnout from an empirical perspective. In a person-centered way, the study found that parental burnout in parents of children with ASD was divided into three subgroups (Low, Medium, and High parental burnout profiles), whereas parental burnout in parents of children without ASD was divided into two subgroups (Low and Medium parental burnout profiles). On one hand, these results indicate that parents of children with ASD are more likely to experience high levels of parental burnout, which is consistent with previous variable-centered findings (Kütük et al., 2021). Meanwhile, the variable-centered results of this study reveal that burnout in parents of children with ASD is twice as severe as that in parents of children without ASD.

On the other hand, this result is different from the previous study suggesting that there were four latent profiles of parental burnout (Suárez et al., 2022). We did not find VHPBP in the Chinese parents’ sample. One possible explanation is the cultural differences, which indicates that parents from individualistic cultures are easier to experience higher burnout (Roskam et al., 2021). Within the collectivist cultural framework prevalent in Eastern societies, parents frequently receive substantial support from extended family networks, particularly grandparents and other relatives in parenting practice. This intergenerational assistance system significantly alleviates parental caregiving burden, thereby effectively mitigating caregiver fatigue and burnout.

Furthermore, the proportion of each latent class provided important information. First, most (84.67%) parents of normal children experienced LPBP and a small proportion of parents (15.33%) experienced MPBP, which may be a general present state reflecting parental burnout in Chinese parents. The heterogeneity of parental burnout was demonstrated from a person-centered perspective, which could be used as a supplement to variable-centered research (Suárez et al., 2022). Second, more than half of the parents raising autistic children experienced MPBP (42.9%) and HPBP (8%). Previous longitudinal studies demonstrated that the initial levels of parental burnout at T1 contribute to higher levels at T2 (Cheng et al., 2020). This indicates that efforts should be made to help parents reduce parental burnout, which could worsen over time. Third, the results indicated that a greater proportion of parents raising children with ASD suffer from higher burnout, which is consistent with previous variable-centered research results (Kütük et al., 2021). These results also support the need to identify the latent profiles of parental burnout in the two parent groups separately.

### Sociodemographic characteristics for different profiles of parental burnout

This study explored the differences in sociodemographic characteristics within each subgroup. The results showed that the child’s gender had no significant effect on the latent class of burnout, indicating that these subgroups of burnout did not differ significantly according to the child’s gender. Children’s age had a significant impact. The younger the child (aged 3–6), the easier it was for the parents to be in a state of HPBP. One possible explanation is that young children are less independent, and parents need to spend most of their time and energy caring for them (Mira et al., 2022). Mothers were the main caregivers of children with ASD (Berenguer et al., 2024), and the accumulation of long-term care burden made mothers more likely to experience HPBP, which is consistent with previous research results (Ren et al., 2024). From the perspective of social division of labor, this is not difficult to understand. While for parents of typically developing children, no significant difference has been found in the subgroups owing to parental identity and role. It may be related to the fact that Chinese families are dominated by nuclear families, and fathers spend more time involving in upbringing their children (Gao et al., 2020; Wang et al., 2022).

Besides, researchers suggest that raising children with severe ASD, having two or more children, and caring for children aged 3–6 are more likely to experience HPBP. Researchers have found that the severity of ASD closely keep up with parenting stress and burnout (Ardic and Olcay, 2021); and poor independence of young children aggravates stress and burden (Zaidman-Zait et al., 2014). While education levels of the parents’ and monthly family income had no significant impact on the class of burnout in parents raising children with ASD. In the context of Chinese culture (Wang and Wang, 2021), extended family networks often provide additional support to reduce the financial and emotional burden on individual parents. They offer valuable parenting expertise and, in some cases, extend financial assistance to support child-rearing efforts (especially for family of children with disabilities). This cultural dynamic may explain why education level and income did not emerge as significant predictors in our study, unlike in studies conducted in more individualistic societies.

### Implications and limitations

The results reveal the heterogeneous characteristics of burnout between the two groups of parents, which could provide a scientific basis for interventions for burnout. First, providing more measures to parents raising children with ASD can help to alleviate parenting stress, preventing them from experiencing higher levels of burnout, as its negative consequences on children’s development have been well-documented (Yan et al., 2024; Yang et al., 2021). Lower levels of burnout mean a better environment for the child’s growth. Fortunately, multiple departments of the current Chinese government are carrying out care initiatives for autistic children, as well as their families, and have issued the “*Implementation Plan for the Promotion of Care for Children with Autism (2024–2028)*.” One of the measures is to provide families of autistic children with more social resources and support.

Second, developing individualized burnout interventions for different categories of parents can precisely meet the needs of diverse groups (Ren et al., 2024). For parents in HPBP, it may be more essential to offer respite services, conduct both directive and non-directive group programs for parental burnout, and provide psychological counseling. For those in MPBP, supplying them with scientific knowledge about autism, home-based ASD intervention methods, and guidance on family education could be beneficial. As for the LPBP, assisting them in identifying stress, discovering coping resources, and learning relaxation skills might prove helpful.

Third, mothers, parents of younger children, those having two or more children, and those having children with severe ASD are particularly susceptible to high levels of parental burnout. As community workers, rehabilitation institution members, and special education teachers, should pay more attention to these families, offering them social support and parenting assistance to help alleviate their stressful situations and reduce burnout.

This study had some limitations. First, although this study had a theoretical basis for investigating related variables, it was still a study within cross-sectional data. Longitudinal investigation should observe trajectory changes through latent transition analysis to better understand the process of parental burnout in future study (Wang et al., 2022). Second, the study is specific to Chinese culture and may not be generalizable across cultures. Previous research has shown that parents in collectivist countries experience lower levels of parental burnout compared to those in individualist countries (Sim et al., 2016). Cross-culture studies on parental burnout are encouraged in future study. Third, we acknowledge that the comparison group may include children with undiagnosed or co-occurring conditions, as we did not screen for conditions such as ADHD, learning disabilities, or other neurological or psychiatric concerns. Besides, it is an important limitation of this study that burnout was measured using only one scale. Multiple data sources, such as experimental methods, observation methods, and evaluations are encouraged in future study. Lastly, we did not collect data on whether families had a psychiatric diagnosis or received treatment. These factors may have a significant limitation given the potential impact of these factors on parental burnout. For future studies, this information should be collected to better understand its role and control for potential confounding effects.

## Conclusion

Three latent parental burnout profiles (LPBP, MPBP, and HPBP) were identified in Chinese parents of children with ASD, while two latent parental burnout profiles (LPBP and MPBP) were identified in Chinese parents of children without ASD. In the overall sample, over 50% of the parents raising children with ASD were found to have MPBP or HPBP. Moreover, mothers, parents of children with severe ASD, those with two or more children, and those with children aged 3–6 years were at a higher risk of experiencing HPBP. This study highlights an urgent need to provide targeted interventions for different subgroups of burnout in parents raising children with ASD.

## Data Availability

The raw data supporting the conclusions of this article will be made available by the authors, without undue reservation.
